# Relationship between Hepatitis B Viral Deoxyribonucleic Acid Load and Hepatocellular Carcinoma

**DOI:** 10.5005/jp-journals-10018-1228

**Published:** 2017-05-05

**Authors:** Muhammad M Hussain, Mamun Al Mahtab, Shahidul Islam, Nooruddin Ahmed, Salimur Rahman, Mobin Khan

**Affiliations:** 1 Department of Hepatology, Bangabandhu Sheikh Mujib Medical University, Dhaka, Bangladesh,; 2 Department of Hepatology, Bangabandhu Sheikh Mujib Medical University, Dhaka, Bangladesh,; 3 Department of Oncology Bangabandhu Sheikh Mujib Medical University, Dhaka, Bangladesh; 4 Department of Hepatology, Bangabandhu Sheikh Mujib Medical University, Dhaka, Bangladesh,; 5 Department of Hepatology, Bangabandhu Sheikh Mujib Medical University, Dhaka, Bangladesh,; 6 Department of Hepatology, Bangabandhu Sheikh Mujib Medical University, Dhaka, Bangladesh,

**Keywords:** Alpha fetoprotein, Fine needle aspiration cytology, Hepatitis B, Hepatitis B surface antigen, Hepatitis B virus deoxyribonucleic acid (polymerase chain reaction), Hepatocellular carcinoma, Ultrasonography.

## Abstract

**Introduction:**

Hepatitis B virus (HBV) infection is an established cause of hepatocellular carcinoma (HCC) and is associated with poor prognosis. High HBV deoxyribonucleic acid (DNA) load has been identified in HCC and hepatitis B surface antigen-positive patients.

**Materials and methods:**

This study was done in the Department of Hepatology, Bangabandhu Sheikh Mujib Medical University, Dhaka, Bangladesh, from January 2006 to December 2007. Thirty patients with HBV infection-related HCC were enrolled. Another 30 patients with HBV-related liver diseases without HCC were analyzed as controls.

**Results:**

The HCC patients had a high viral load (>10^5^ copies/mL), while all of the controls had low (<10^5^ copies/mL) viral load.

**Conclusion:**

It seems that patients with HCC bear high HBV DNA loads in Bangladesh, but the causes underlying this remain to be resolved.

**How to cite this article:** Hussain MM, Al Mahtab M, Islam S, Ahmed N, Rahman S, Khan M. Relationship between Hepatitis B Viral Deoxyribonucleic Acid Load and Hepatocellular Carcinoma. Euroasian J Hepato-Gastroenterol 2017;7(1):111-112.

## INTRODUCTION

Hepatitis B virus (HBV) infection is an established cause of hepatocellular carcinoma (HCC) and is associated with poor prognosis.^1^ Vast majority of this disease burden occurs in developing countries. Although several risk factors have been exposed about HBV-related HCC, these vary considerably in different parts of the world. However, high viral load remains as one of the important risk factors.^2^ In this preliminary study at Bangladesh, we opted to assess if HBV deoxyribonucleic acid (DNA) levels remain as a critical factor of HBV-related HCC patients.

## MATERIALS AND METHODS

The study was conducted at Bangabandhu Sheikh Mujib Medical University, Dhaka, during 2 years (2006 and 2007) in a total of 60 patients in which 30 had HBV-related HCC and 30 patients had chronic HBV-related liver diseases but no HCC. The HBV DNA was extracted from sera using HBV Real-TM Quant (Sacace Biotechnologies srl., Italy) following the manufacturer’s instructions.

Data were checked for any possible error and were analyzed by Statistical Package for the Social Sciences program. Significance of the test was tested by Student’s t-test and chi-squared test (χ^2^). Result of test was statistically significant if p-value < 0.05.

## RESULTS AND DISCUSSION

The mean age of the cases with HCC was higher (44.5 × 12.4 years) than that of their control counterparts (37.7 × 15.1 years); however, the difference between the two groups was not statistically significant (p = 0.064). A male preponderance was observed in both cases and controls. All of the HCC patients exhibited an alpha-fetoprotein level of >400 ng/mL, whereas none of the control group patient had alpha-fetoprotein above this level (p < 0.001). All of the patients with HCC had HBV DNA load >10^5^ copies/mL, while all 30 patients of control group without HCC had HBV DNA load 10^5^ or below 10^5^ copies/mL (p < 0.001).

In HBV-related liver disease patients, HBV DNA is one of the strongest predictive factor for the development of HCC. The results of this study were compatible with those^1,2^ from India, a neighboring country of Bangladesh. It has been reported that patients of chronic HBV infection followed by chronic hepatitis B virus (HCV) infection were at higher risk of developing HCC in India. Chronic alcohol consumption was found to be a risk factor in cirrhotic cases only when it was associated with HCV ribonucleic acid positivity. Most of the patients had a large tumor size (>5 cm) with multiple liver nodules, indicating an advanced stage of the disease, thus making curative therapies difficult.^3^ However, this is a preliminary study and other factors related to HCC development in HBV infection, such as HCV infection or history of alcohol consumption have not been assessed here. As it has been mentioned that HCC can be delayed by use of antiviral drugs, it appears that antiviral drugs may be used in patients with pre-HCC stages in Bangladesh.^4^
